# Editorial: Modern advances in arthroplasty

**DOI:** 10.3389/fsurg.2025.1568587

**Published:** 2025-02-26

**Authors:** Jason Werle, Mansour Abolghasemian

**Affiliations:** ^1^ Division of Orthopedic Surgery, Department of Surgery, University of Calgary, Calgary, AB, Canada; ^2^ Division of Orthopedic Surgery, Department of Surgery, University of Alberta, Edmonton, AB, Canada

**Keywords:** arthroplasty, robotics, augmented/virtual reality AR/VR, artificial intelligence, navigation, 3D printing, patient specific instrumentation

**Editorial on the Research Topic**
Modern advances in arthroplasty

The integration of cutting-edge technologies has led to profound advancements in arthroplasty in recent years. These developments aim to optimize surgical precision, enhance patient outcomes, and reduce complications. This article explores key technological advances in arthroplasty and their impact on the future of orthopedics.

## Robotic-assisted arthroplasty

Robotic-assisted surgery has emerged as a transformative technology in joint reconstruction, particularly in total knee arthroplasty (TKA) and total hip arthroplasty (THA). Robot technology can enhance precision in bone preparation, implant alignment, and soft tissue balancing (1). Recent studies suggest robotic-assisted TKA improves alignment, reduces outliers, and may enhance function and implant longevity (2).

The integration of advanced imaging, such as computed tomography (CT) scans or Magnetic Resonance Imaging (MRI), allows for detailed anatomical mapping, enabling surgeons to create individualized surgical plans. Intraoperative sensors further refine this process by providing real-time feedback (3). While short-term outcomes are promising, long-term studies are needed to confirm the durability and clinical superiority of robotic-assisted arthroplasty.

Initially limited by cost, complexity, and longer surgeries, robotic adoption is expanding with affordable, user-friendly platforms. The development of systems that eliminate the need for CT or MRI while enhancing rotational alignment precision is expected to drive broader adoption. Their use encourages physiologic alignments that require precise cuts, offering another potential advantage (4).

## Artificial intelligence and machine learning

Artificial intelligence (AI) and machine learning are becoming integral to modern medicine. These technologies analyze large datasets to identify patterns and make predictions, offering insights into patient outcomes, implant survivorship, and surgical complications (5). AI-powered algorithms can assist in patient selection, preoperative planning, and postoperative care (6).

One promising application is the prediction of prosthetic joint infections (7) and aseptic loosening (8) using patient-specific risk factors and perioperative data. Furthermore, AI-driven tools are being developed to optimize implant selection and placement by analyzing biomechanical forces and patient anatomy (9). These advancements could lead to more personalized treatments.

While AI has tremendous potential, challenges remain in integrating these tools into clinical practice. Issues such as data stewardship and privacy, algorithm transparency, and clinician training must be addressed to maximize the utility of AI in arthroplasty.

## Advances in biomaterials

Innovations in biomaterials have revolutionized arthroplasty by enhancing implant durability and biocompatibility. Highly cross-linked polyethylene (HXLPE) has significantly reduced wear rates in THA and TKA, minimizing the risk of osteolysis and aseptic loosening (10). Similarly, ceramics and advanced metal alloys offer superior strength and corrosion resistance, extending implant longevity (11). Advanced metal-bearing technologies have rekindled interest in hip resurfacing for select patients, enhancing functional outcomes while maintaining implant durability (12).

Bioactive coatings, such as hydroxyapatite and titanium plasma spray, improve osseointegration and reduce the risk of implant failure (13). Additionally, antimicrobial coatings are being developed to combat prosthetic joint infections (PJIs), a devastating complication of arthroplasty. Silver, iodine, and antibiotic-releasing coatings are promising innovations in this domain, but require further research (14).

## Enhanced perioperative techniques

Recent advances have improved perioperative management in arthroplasty. Enhanced recovery after surgery (ERAS) protocols, incorporating multimodal pain management, minimally invasive techniques, and rapid rehabilitation, have become standard in many centers. These protocols leverage technology to reduce hospital stays, lower costs, and improve patient satisfaction (15).

Medial pivot knee designs mimicking the natural kinematics of the knee with a stable medial compartment and a more mobile lateral compartment has the potential to improve patient outcomes and reduce wear, contributing to better implant longevity (16).

Navigation systems and computer-assisted surgery (CAS) further enhance perioperative precision. These tools assist in achieving optimal alignment and positioning of implants, critical for long-term success (17). CAS has been suggested to reduce malalignment and improve outcomes in TKA, though its cost-effectiveness remains debated (18). The future of CAS with the advent of Robotic-Assisted Arthroplasty will have to be determined.

Patient-specific instrumentation (PSI) enables surgeons to use preoperative imaging data to create custom surgical guides. While PSI has been associated with improved accuracy and reduced operative times, its widespread use is limited by high costs and time-consuming production processes (19). The emergence of robotics may also limit its future role.

The development of 3D-printed implants represents another groundbreaking advancement. Currently, 3D printing is primarily applied in complex revision arthroplasty for patients with severe bone loss, enabling the customization of implants to match unique anatomy (20). However, the use of 3D printing for primary implant manufacturing processes is expanding. Integrating AI and 3D printing could revolutionize arthroplasty, driving the field toward precision medicine (21).

## Augmented- and virtual reality

Augmented reality (AR) and virtual reality (VR) are emerging technologies with the potential to transform arthroplasty performance and education. AR overlays digital information onto the surgical field, providing real-time guidance for implant placement and alignment (22). VR, on the other hand, offers immersive surgical training and preoperative planning tools (23).

Early studies suggest that AR can enhance surgical accuracy and reduce complications (24). VR-based training has been shown to improve surgical skills and confidence, particularly among novice surgeons (25). As these technologies evolve, they may become integral components of arthroplasty practice and education.

## Perspectives and future directions

The rapid pace of technological advancements in arthroplasty holds immense promise, but challenges remain. Cost-effectiveness is a significant barrier, particularly for resource-limited settings. Developing affordable and scalable technologies will be crucial to ensuring equitable access. Rigorous clinical studies are needed to validate the long-term efficacy and safety of these innovations. While many technologies show promise in early studies, their impact on implant survivorship, patient-reported outcomes, and overall healthcare costs requires longer-term investigation.

Interdisciplinary collaboration between engineers, clinicians, and data scientists will be essential to drive innovation in arthroplasty. Regulatory frameworks must also evolve to address the unique challenges posed by new technologies, such as AI and 3D printing.

In conclusion, modern technological advances are revolutionizing arthroplasty, offering new opportunities to improve patient care. While challenges remain, the integration of robotics, AI, advanced biomaterials, and immersive technologies represents a paradigm shift in joint replacement surgery. With continued research and collaboration, these innovations have the potential to redefine the future of arthroplasty surgery.
